# pyHIVE, a health-related image visualization and engineering system using Python

**DOI:** 10.1186/s12859-018-2477-7

**Published:** 2018-11-26

**Authors:** Ruochi Zhang, Ruixue Zhao, Xinyang Zhao, Di Wu, Weiwei Zheng, Xin Feng, Fengfeng Zhou

**Affiliations:** 1 BioKnow Health Informatics Lab, College of Computer Science and Technology, and Key Laboratory of Symbolic Computation and Knowledge Engineering of Ministry of Education, Jilin University, Changchun, 130012 Jilin China; 2 BioKnow Health Informatics Lab, College of Software, Jilin University, Changchun, 130012 Jilin China

## Abstract

**Background:**

Imaging is one of the major biomedical technologies to investigate the status of a living object. But the biomedical image based data mining problem requires extensive knowledge across multiple disciplinaries, e.g. biology, mathematics and computer science, etc.

**Results:**

pyHIVE (a Health-related Image Visualization and Engineering system using Python) was implemented as an image processing system, providing five widely used image feature engineering algorithms. A standard binary classification pipeline was also provided to help researchers build data models immediately after the data is collected. pyHIVE may calculate five widely-used image feature engineering algorithms efficiently using multiple computing cores, and also featured the modules of Principal Component Analysis (PCA) based preprocessing and normalization.

**Conclusions:**

The demonstrative example shows that the image features generated by pyHIVE achieved very good classification performances based on the gastrointestinal endoscopic images. This system pyHIVE and the demonstrative example are freely available and maintained at http://www.healthinformaticslab.org/supp/resources.php.

## Background

Besides OMIC data, imaging is another major source of biomedical information for the biomedical modeling [1]. And imaging has its inherent nature of non-invasively and instantly monitoring the health status inside the body [2], while the OMIC data is produced hours or longer later after the sample is collected. So imaging and OMIC data represent different modalities and resolution of a biological system [3].

Biomedical images have already been widely used in the diagnosis and prognosis modeling [4–6]. The image histogram of oriented gradients (HOG) feature was used for the predictions of lung cancers [7]. And the rotation-invariant local binary pattern (LBP) feature and its variants were utilized to investigate the texture and other image patterns in biomedical images [8–10]. Other image features like the gray-level co-occurrence matrix (GLCM) were also frequently utilized in predicting the tumor outcomes and other phenotypes [11]. Image segmentation, denoising and fractal image features were essential to improve the image based classification problems [4–6].

This work presented a user-friendly system, pyHIVE, to extract five widely used image features using the Python programming language. The existing image feature extraction softwares usually focus on one algorithm, and the users need to implement a separate script to optimize the prediction performance of a disease classification model. pyHIVE closes the gap between the raw biomedical images and the standard input for the data mining researchers. pyHIVE provides five widely used image feature extraction algorithms and produces all the data matrices required for a standard data mining task. A demonstrative script is also provided for a PCA-based feature selection and binary classification example. A parallelization function is implemented for a better utilization of the widely-used multi-core computing environments. We hope that pyHIVE may help the researchers of interest intuitively ask data mining questions from the biomedical images, and facilitate the faster developments of image-based disease diagnosis and prognosis modeling.

## Materials and methods

### Dataset details

We demonstrated how to use pyHIVE by a public dataset of gastrointestinal endoscopic images. The endoscopic images are publicly available at the El Salvador Atlas of Gastrointestinal Video Endoscopy, as similar in [12]. 243 images were randomly captured from the 16 endoscopic videos of normal participants, and each is 1280 × 720 pixels in size. These were regarded as the negative images. There are 17 gastric polyp videos, 26 gastric ulcer videos and 10 gastritis videos available at the database. A random image capturing step generated 158, 99 and 74 images from these three groups of videos, respectively. These 331 (=158 + 99 + 74) images were regarded as the positive images, and are 352 × 240 pixels in size. The negative images were scaled to the same size of the positive ones, which is a user-defined parameter of pyHIVE.

### Workflow and explanations of the pyHIVE code

**Fig. 1:**
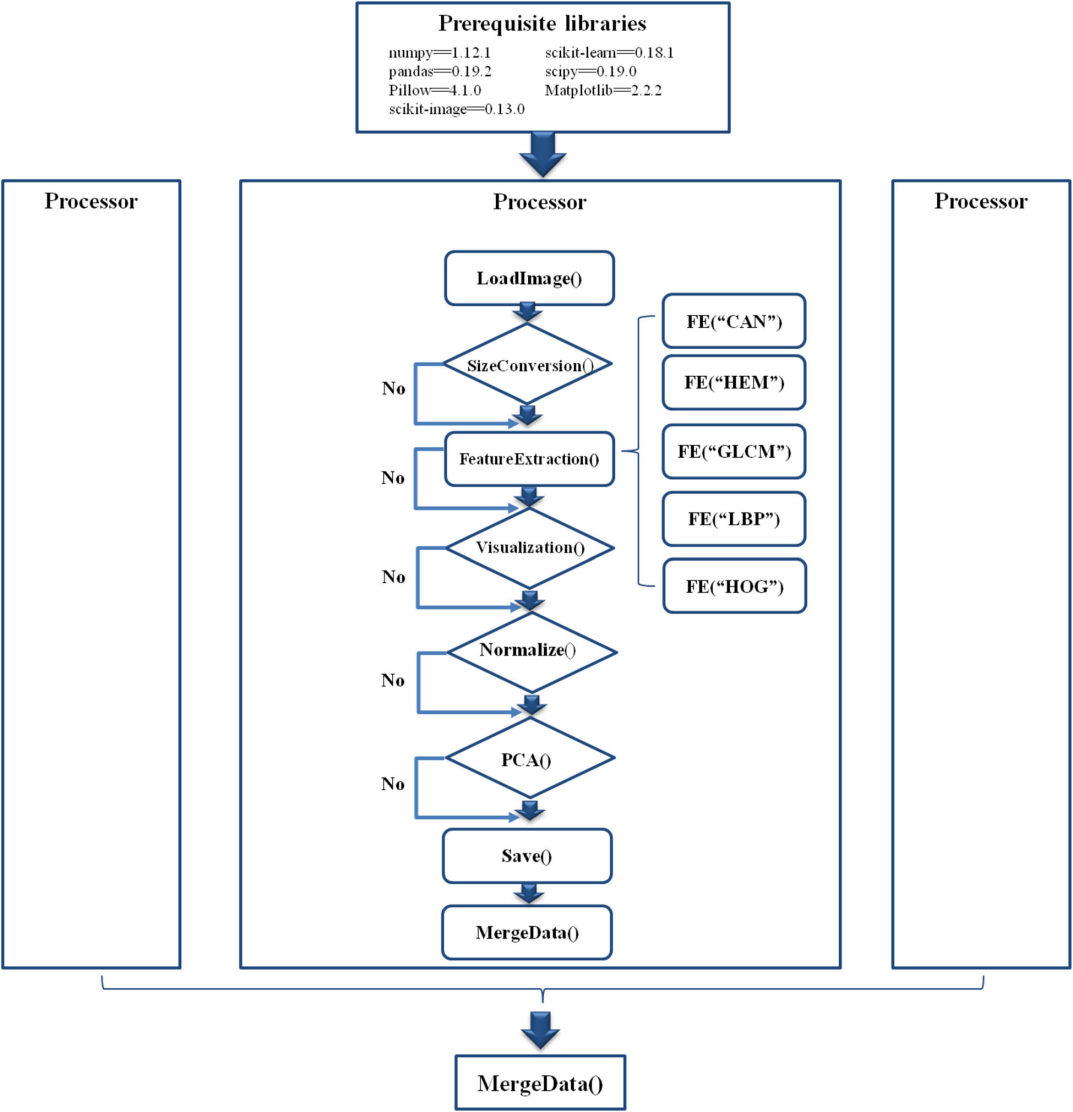
Work flow of pyHIVE. The calculations are distributed to different CPU processor, and each Processor module does the same task for different images. All the feature choices and parameters may be tuned in the configuration file provided by the users

The software pyHIVE was designed as a parallel image feature engineering system, and the overall workflow was demonstrated in Fig. 1. Firstly, pyHIVE has a few prerequisite python packages, including numpy version 1.12.1, pandas version 0.19.2, Pillow version 4.1.0, scikit-image version 0.13., scikit-leran version 0.18.1, and scipy version 0.19.0. These python packages were pre-installed in many python distributions. But in case that these python packages were not installed, the user may manually install them using the python command “pip”, e.g., pip install numpy.

After all the prerequisite python packages were available, the user may run pyHIVE by the command line “python main.py”. pyHIVE loads all the image files in the sub-directory “images” by default, and the output files will be in the sub-directory “features”. All the parameters of the five image feature engineering algorithms may be tuned in the configuration file “config.cof”. Default values for all the parameters are also provided for the convenience of the users.

A task “Processor” will be executed on an input image file and image features will be calculated based on the user-specified algorithms and their parameters. An optional “Normalize” step is provided. All the calculated features will merged into one file for each image file. pyHIVE will parallelly run multiple “Processor” tasks based on the user-specified parameter “njob” in the configuration file config.cof. Feature files of all the images will be merged into one matrix file.

## Implementation

pyHIVE implemented five widely-used image feature extraction algorithms, i.e. Histogram of Oriented Gradient (HOG), Local Binary Pattern (LBP), Gray-level Co-occurrence Matrix (GLCM), Hessian Matrix (HEM) and Canny (CAN), using the programming language Python. HOG, LBP and GLCM are three widely-used image features to describe textures [7–9, 11]. HEM and CAN work well on object topology detections in images [13, 14]. A post-processing module using Principal Component Analysis (PCA) was also provided. The eigenvalues of the aforementioned five algorithms generate different value scales, and the user may choose to normalize the data using an embedded normalization module.

pyHIVE also parallelized the calculations of the aforementioned image feature extraction algorithms. The calculation of image features has an inherent nature of O(*n* × *m*) time complexity, where *n* and *m* are the width and height of an image, respectively. Some image feature extraction algorithms have been implemented using interpretive languages like MatLab, and may run for minutes or even hours to extract features of a large image. It may become intolerable in real-time analysis situations or for thousands of biomedical images. Python was chosen to implement pyHIVE for its fast running and a large repository of data mining modules. pyHIVE may fully utilize the computing power of multi-core architecture of the modern servers. Due to that the Windows operation system does not provide a strong support for the parallel programming interface in Python, the Windows version of pyHIVE does not support parallelized calculation. It has a time cost to switch between computing processes in the same CPU core, so it is recommended that the number of parallel tasks may be set to the number of CPU cores in the user server.

### Extensive support for input and output formats

pyHIVE has been conceived and implemented as a user-friendly image feature extraction system. So pyHIVE has been tested using both Python v2 and Python v3 in all the three main operation systems, i.e. Linux, Mac OS and Windows. pyHIVE accepts 30+ image file formats as input, e.g. BMP, EPS, GIF, JPG, PNG, PPM, and SGI, etc. The calculated features may be saved as one or more choices of the six supported file formats, i.e. CSV, PICKLE, JSON, EXCEL, TXT and SQL. The user may decide the number of fractional digits of the decimal values of the calculated image features. A larger file size and a longer saving time will be needed if a larger number of fractional digits is chosen.

Due to the many parameters that a user may want to manipulate, pyHIVE defined a configuration file to help the user tune how pyHIVE works for different experiment requirements. All the aforementioned functionalities may be tuned by parameters in the configuration file. A default value was also provided for these parameters, so that a user may run pyHIVE with a minimum parameters.

## Results

We demonstrated how to use pyHIVE by a public dataset of gastrointestinal endoscopic images. The endoscopic images are publicly available at the El Salvador Atlas of Gastrointestinal Video Endoscopy, as similar in [12]. Detailed description of the dataset may be found in the section “Dataset details”. Two feature extraction algorithms HOG and LBP were selected to investigate the above binary classification problem. HOG and LBP generated 95,256 and 84,480 features for each of the images, respectively. In order to avoid the over-fitting problem, the PCA module provided by pyHIVE was applied to the feature matrix. The default number of principal components was the smaller one of the two numbers of samples and features. So the number of features was reduced to 574 (=243 + 331). Five representative classifiers, i.e. Support Vector Machine (SVM), Nearest Neighbor (NN), Decision Tree (DTree), Naïve Bayes (NBayes), Logistic Regression (LR), and Random Forest (RF), were chosen to evaluate the classification performance of a selected feature subset, as described in [15]. Accuracy was defined as the percentage of correctly predicted images, and the maximum accuracy (mAcc) of the five classifiers based on the given feature subset was defined as the performance measurement. All the classification performance measurements were calculated by the stratified k-fold cross validation strategy with the parameter *k* = 10 by default.

**Fig. 2:**
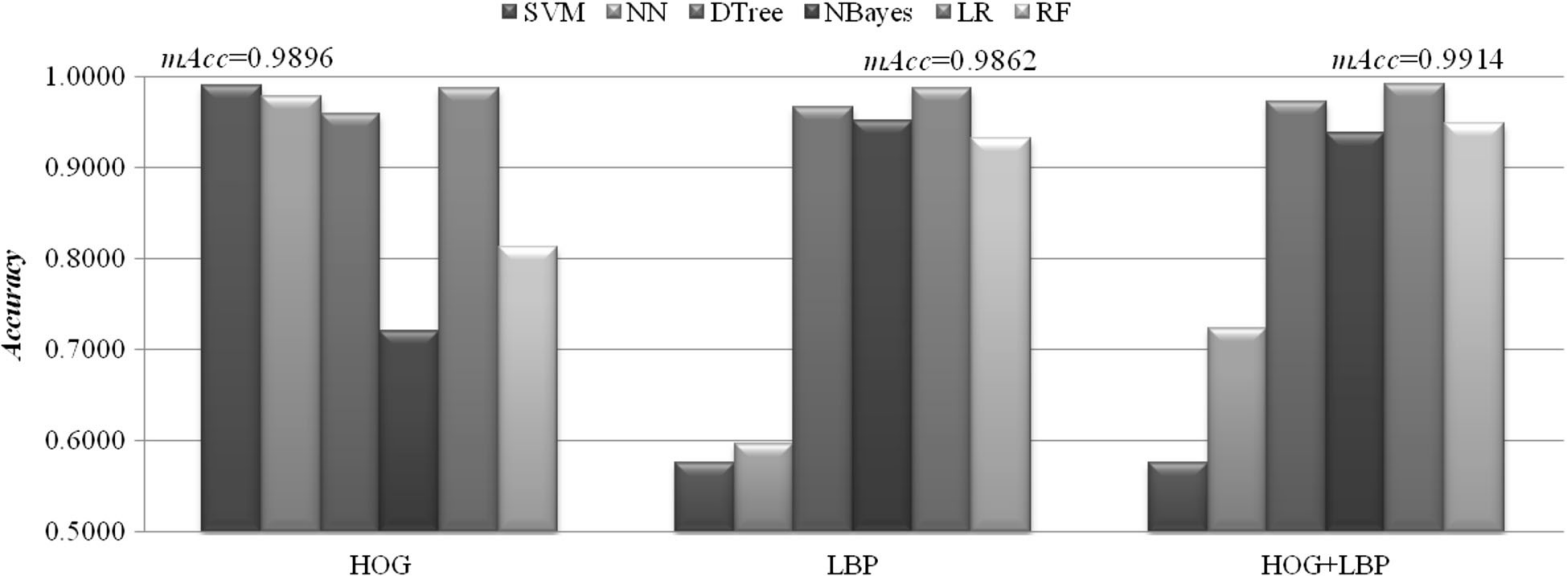
Binary classification accuracy of the HOG and LBP image features generated by pyHIVE. mAcc is the maximum accuracy of the five classifiers

After the preprocessing of PCA, both HOG and LBP features produced accurate classification models, as shown in Fig. 2. The feature scales of HOG and LBP are significantly different from each other. So they were processed using the normalization module provided by pyHIVE. HOG generated the best model with mAcc = 0.9896, and SVM performed the best among the five classifiers. It is interesting to observe that the second best model was achieved by LR with Accuracy = 0.9861. The LBP features worked best with the classifier LR, and achieved mAcc = 0.9862. SVM didn’t work well with the LBP features, and only achieved Accuracy = 0.5760. A further improvement was achieved by combining features from both HOG and LBP, and the best model of mAcc = 0.9914 was achieved using the classifier LR. So generally, the classifier LR works very well with the image features generated by the HOG and LBP algorithms.

**Fig. 3:**
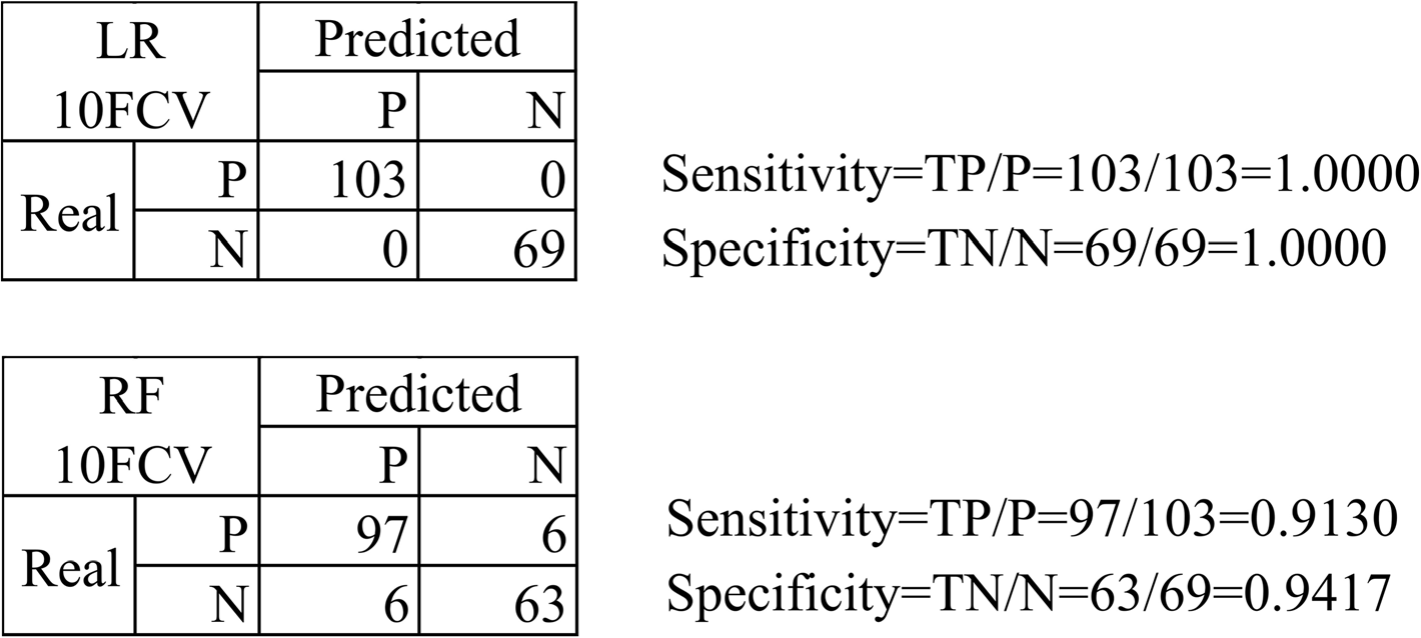
Classification accuracies for the individual classes by the classifiers LR and RF. The left matrix was the confusion matrix of the 10-fold cross validation performances of the classifiers LR and RF. Sensitivity was the prediction accuracy of the positive samples, while the specificity was the accuracy of the negative samples

The classification performances of individual classes were evaluated for the best classifier LR, as shown in Fig. 3. The previous paragraph evaluated ten classifiers on three feature sets. So it’s difficult to demonstrate all the confusion matrices and the prediction accuracies of the individual classes. The whole dataset of the features HOG+LBP was randomly split into the training (70%) and test datasets. The confusion matrix of the best classifier LR was calculated by the 10-fold cross validation strategy, as illustrated in Fig. 3. Both P and N classes were perfectly predicted, with sensitivity = 1.0000 and specificity = 1.0000. The experimental data suggested that besides the overall accuracy, the individual classes were also accurately predicted. Another classifier RF didn’t perform as well as LR, and only achieved 0.9130 and 0.9417 for sensitivity and specificity, respectively.

Another evaluation experiment of pyHIVE was carried out on the two classes of canvas and cloth texture images from Contrib_TC_00000 of the Outex Texture Database [16]. Each class has 42 images and all the images are 256 × 256 in pixels. Two feature engineering algorithms GLCM and HEM were chosen for pyHIVE, and all the parameters used the default values. These two classes of texture images were significantly different from the endoscopic images, and may demonstrate pyHIVE’s generalization on different image styles. A binary classification model was trained and evaluated, as described above. The classifier RF achieved worst accuracy 0.92 based on the GLCM features, while the other five classifiers achieved 1.00 in accuracy. All the classifiers except for LG and KNN achieved 1.00 in accuracy based on the HEM features. So the pyHIVE-generated image features perform well on the texture images, too.

**Fig. 4:**
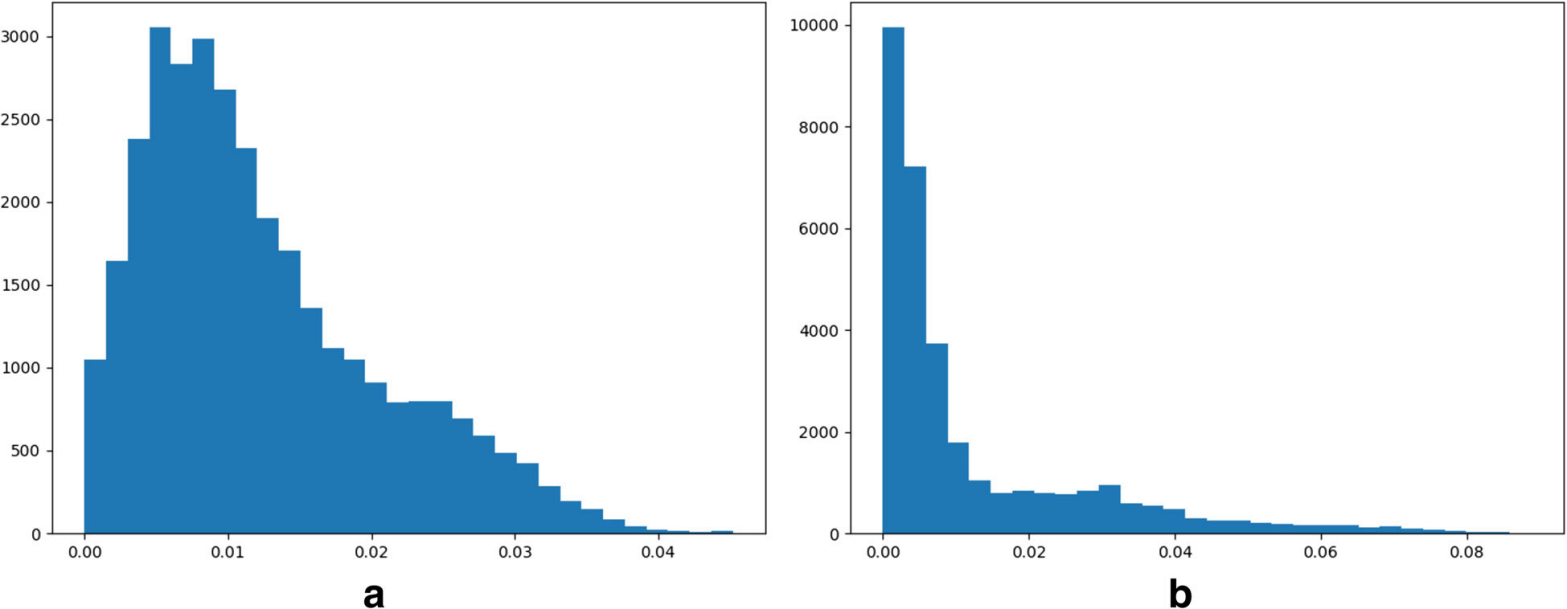
Feature histograms calculated by pyHIVE. The non-normalized HOG features were calculated for (**a**) a canvas image 007744 and (**b**) a cloth image 007751

The features of a given image file may be visualized as a histogram, as shown in Fig. 4. The algorithm HOG was chosen to calculate the features for two images 007744 and 007751. The two images belong to the classes of canvas and cloth, respectively. So these two classes of texture images may be visually inspected for the differences between their HOG features. It is anticipatable to have a high accuracy for the binary classification model between these two classes of texture images.

## Discussion

This work proposed a biomedical-image feature extraction system pyHIVE, and an easy-to-use binary classification pipeline. Biomedical imaging technology produces huge amount of data, and requires specialized skills to read and analyze. The proposed system pyHIVE can generate a standard feature matrix from the biomedical images, so that a machine learning scientist may investigate the modeling part directly without the sophisticated imaging feature extraction knowledge. Five widely used image feature extraction algorithms were implemented and pyHIVE accepts 30+ input image formats. The generated data matrix may be saved as one of the six standard file formats, i.e. CSV, PICKLE, JSON, EXCEL, TXT and SQL. To facilitate a quick prototyping investigation, a standard binary classification pipeline is also provided.

The parallelization performance of pyHIVE was tested in an Inspur Gene Server G100 with 256GB memory, 28 Intel Xeon® CPU cores (2.4GHz), and 26 TB hard disk. When pyHIVE ran under a single process, it spends 17.1667 s in extracting features from 574 images. When pyHIVE used 28 processes, only 1.2379 s was used to extract features from the aforementioned images. The result shows that the use of multi-cores computer can effectively reduce the time of image feature extraction, approximately 13.8676 times faster than the single-process version. So the parallelization module significantly increased the running speed of pyHIVE.

## Conclusions

The python package pyHIVE proposed in this study is an easy-to-use biomedical image feature extraction system, and quite a few functional modules are also provided to facilitate a fast and intuitive exploration of an image based classification dataset. A large number of image file formats may be fed to pyHIVE, and the results may be exported as various standard file formats. pyHIVE is anticipated to help the biomedical image based research studies.

## Abbreviations

CAN: Canny
DTree: Decision Tree
GLCM: Gray-level co-occurrence matrix
HEM: Hessian Matrix
HOG: Histogram of oriented gradients
LBP: Local binary pattern
LR: Logistic Regression
mAcc: Maximum accuracy
NBayes: Naïve Bayes
NN: Nearest Neighbor
PCA: Principal Component Analysis
pyHIVE: Python-based Health-related Image Visualization and Engineering system
RF: Random Forest
SVM: Support Vector Machine
